# Case report: Autoimmune nodopathy with concurrent serum and CSF IgG4 anti-neurofascin 155 antibodies

**DOI:** 10.3389/fimmu.2022.1028282

**Published:** 2022-09-30

**Authors:** Wanyu Wang, Lingchun Liu, Mingzhi Zhang, Ruihan Yang, Da Liu, Shunyu Yang, Qiang Meng

**Affiliations:** ^1^ Department of Neurology, The Affiliated Hospital of Kunming University of Science and Technology, The First People’s Hospital of Yunnan Province, Kunming, China; ^2^ Department of Neurology, The First People’s Hospital of Yunnan Province, The Affiliated Hospital of Kunming University of Science and Technology, Kunming, China

**Keywords:** autoimmune nodopathy (AN), chronic inflammatory demyelination polyneuropathy (CIDP), anti-neurofascin 155 antibodies, anti-GD1b antibodies, sensory ataxia, case report

## Abstract

**Objective:**

To report a case of autoimmune nodopathy (AN) with concurrent serum and CSF immunoglobulin (Ig)G4 anti-neurofascin 155 (NF155) and anti-GD1b antibodies.

**Methods:**

A 20-year-old male presented distal weakness of the 4 limbs, hypoesthesia, absent tendon reflexes and sensory ataxia. Nerve conduction studies (NCS), MRI, and autoantibody tests were performed.

**Results:**

NCS revealed a diffuse demyelinating neuropathy in the peripheral nerve with motor and sensory involvement. MRI of the cervical and lumbar plexus showed diffuse enlargement. IgG4 anti-NF155 antibodies in both serum and CSF and IgG anti-GD1b antibodies in serum were positive. After treatment with IVIg, rituximab, and plasma exchange, the titer of the patient’s anti-NF155 antibodies decreased, but symptoms did not significantly improve.

**Discussion:**

This patient presented a typical clinical feature of AN with serum and CSF anti-NF155 antibodies and serum anti-GD1b antibodies coexistent but poor response to IVIg, rituximab and plasma exchange. Early detection of antibodies may be helpful in both diagnosis and therapy of the disease. And prospective studies are necessary to demonstrate the potential role of anti-NF155 antibodies in CSF and help further understand this complex and heterogeneous disease.

## Introduction

Chronic inflammatory demyelination polyneuropathy (CIDP) is a clinically and pathologically diverse autoimmune syndrome of the peripheral nervous system, which could cause significant disability (1, 2). Autoantibodies against the node and paranode of Ranvier, such as neurofascin 155 (NF155) (3), have been described in small subsets of patients with CIDP, sharing immunopathologic mechanisms, clinical features, and treatment response, which differ from those of typical CIDP (4, 5). The existence of anti-paranodal axo–glial junctional molecules leads to different morphological features of the peripheral nerves in this group of patients from conventional CIDP patients, characterized by paranodal dissection and the absence of classical macrophage-mediated demyelination (6). This has led to the appearance of the autoimmune nodopathy (AN) diagnostic category in the recent update of the European Academy of Neurology/Peripheral Nerve Society CIDP diagnostic guidelines (7). Herein we report a case of anti-NF155 positive AN presenting with distal weakness, hypoesthesia, absent tendon reflexes and sensory ataxia. Diffuse demyelinating neuropathy and enlargement of cervical and lumbar plexus were found. Especially, IgG4 anti-NF155 antibodies in both serum and CSF and IgG anti-GD1b antibodies in serum were positive.

## Case summary

### Clinical presentation and physical examination

A 20-year-old male with no medical history was admitted to our department with progressive distal muscle weakness and unstable walking for 5 weeks. He had vomiting and diarrhea initially, and 10 days later, weakness developed. Physical examination on the first hospital day (H1) showed distal weakness of the 4 limbs (the muscle strength of the distal limbs was grade 4), hypoesthesia, absent tendon reflexes and sensory ataxia. No muscle atrophy or fasciculation were observed. The modified Rankin Scale (mRS) rated 2 out of 5.

### Clinical findings

**Figure 1:**
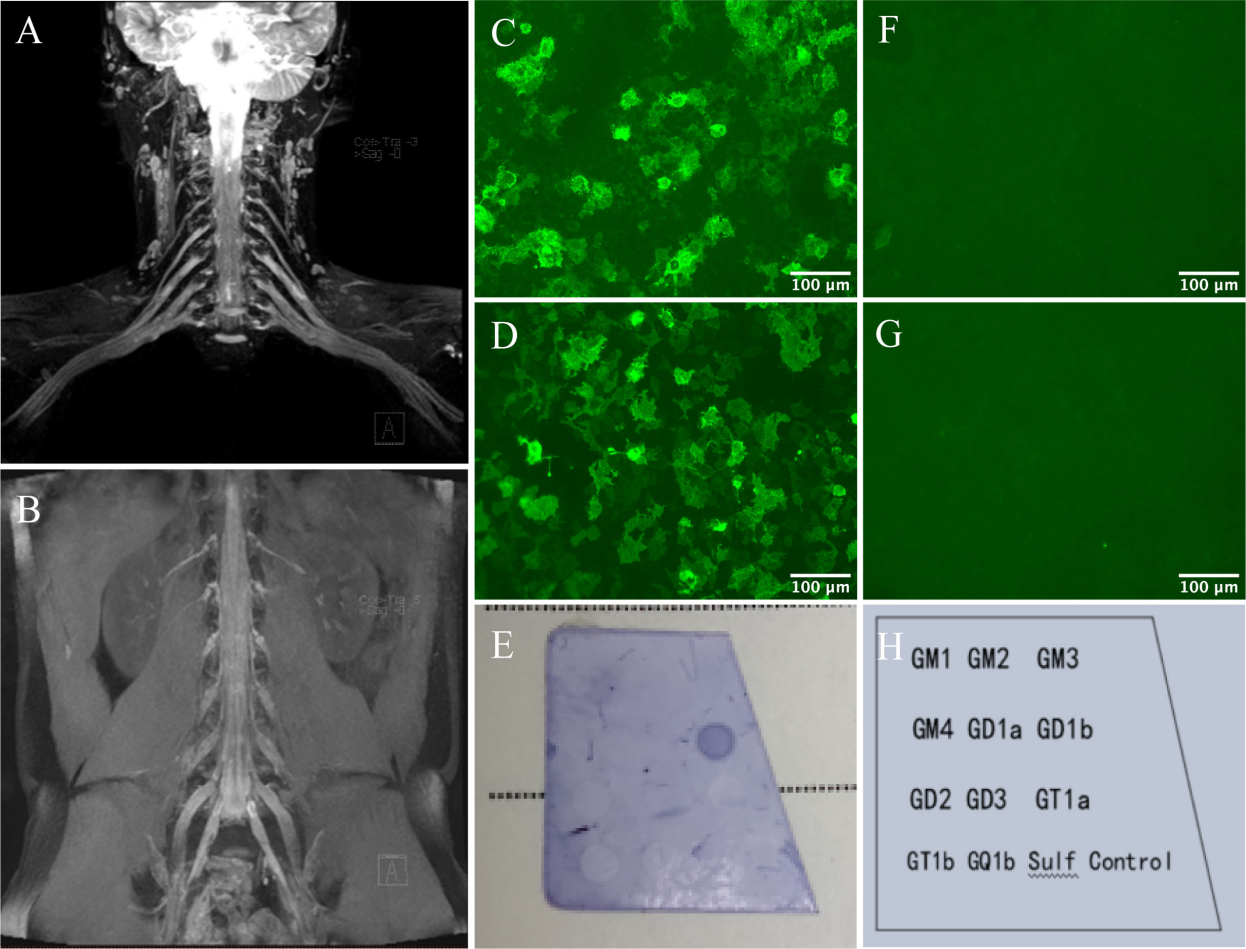
Nerve imaging and autoantibodies against NF155 and GD1b. Symmetrical bilateral enlarged cervical and lumbar plexus on MIP Thin Rage **(A, B)**. The presence of anti-NF155 antibodies was confirmed with NF155-transfected Human Embryonic Kidney (HEK) 293 cells using a cell-based assay (CBA). Reactivity was analyzed by immunofluorescence. Serum **(C)** and CSF **(D)** anti-NF155 antibodies were positive (×400), while negative in the control group of serum **(F)** and CSF **(G)**. Scale bar = 100 μm. The presence of anti-GD1b antibodies was confirmed by dot immunoassay. A clearly discernible color spot was shown in the GD1b antigen-coated area of the test strip **(E, H)**.

Routine blood tests were normal, but serum Epstein-Barr virus (EBV) viral capsid antigen IgG and nuclear antigen IgG antibodies were positive. Nerve conduction studies (NCS) revealed a diffuse demyelinating neuropathy in the peripheral nerve with motor and sensory involvement (**Tables 1**, **2**). Cerebral MRI and visual evoked potential (VEP) were normal. MRI of the cervical and lumbar plexus showed diffuse enlargement (**Figure 1**). CSF analysis showed significantly elevated protein levels (4.178 g/L, average <0.45) and a white blood cell count of 2x10^6^/L (within normal limits). Tests for antibodies showed positive IgG4 anti-NF155 antibodies in both serum and CSF (titers of 1:320 and 1:32, respectively) and seropositive IgG anti-GD1b antibodies (**Figure 1**).

**Table 1:** Sensory nerve conduction.

Sensory nerve	Amplitude, μV	Velocity, m/s
R Ulnar	1.45	24.6
L Ulnar	2.4	24.3
R Radial	1.23	33
L Radial	3.2	31.5
R Median	NR	NR
L Median	3.3	30.9
R Superficial peroneal	NR	NR
L Superficial peroneal	NR	NR
R Sural	NR	NR
L Sural	NR	NR

μV, microvolt; m/s, meter per second; R, right; L, left; NR, not recordable.

Slow velocity and decreased amplitude were found in the bilateral radial, left median, and bilateral ulnar nerve. No response was elicited in the right median and bilateral peroneal and superficial peroneal nerve.

**Table 2:** Motor nerve conduction.

Motor nerve	Sites of Stimulation	Latency, ms	Amplitude, mV	Velocity, m/s	F-Wave Latency, ms
R Ulnar	Wrist-ADM	3.95	15	−	49.5
	B.Elbow	11.3	14.6	38.8	−
L Ulnar	Wrist-ADM	4.23	13.7	−	46.9
	B.Elbow	11.2	11.5	39.5	−
	A.Elbow	14.3	11.3	37.1	−
R Median	Wrist-APB	6.94	10.2	−	51.3
	B.Elbow	14.1	9.5	33.5	−
L Median	Wrist-APB	6.65	7.2	−	52.8
	B.Elbow	13.2	6.7	35.1	−
	A.Elbow	18.1	7.7	36.7	−
R Tibial	Ankle-AH	7.71	6.6	−	NR
	Pop fossa	21.6	3.5	31.0	−
L Tibial	Ankle-AH	8.14	6.0	−	NR
	Pop fossa	20.9	3.2	33.3	−
R Peroneal	Ankle-EDB	10.1	2.7	−	−
	Fib head	20.3	2.6	29.4	−
L Peroneal	Ankle-EDB	10.3	1.78	−	−
	Fib head	20.7	1.29	28.8	−

ms, millisecond; mV, millivolt; m/s, meter per second; R, right; L, left; A.Elbow, above elbow; B.Elbow, below elbow; ADM, abductor digiti minimi; APB, abductor pollicis brevis; AH, abductor hallucis; Pop fossa, popliteal fossa; EDB, extensor digitorum; Fib head, fibular head; NR, not recordable.

Increased latencies, slow velocity, and normal amplitude were observed in the bilateral median, bilateral tibial, and left ulnar nerve. The right ulnar nerve had a slow velocity, normal latency, and normal amplitude. Increased latencies, slow velocity, and reduced amplitude were observed in the bilateral common peroneal nerve. The compound muscle action potentials were measured from peak to peak. Delayed F-wave was observed in the bilateral median and bilateral ulnar nerve. No F-waves were elicited from the bilateral tibial nerve.

### Diagnosis and treatment outcome

This patient was initially diagnosed with Guillain-Barré Syndrome. A three-day course of IVIg (0.4 g/kg·d) started on H3. However, he responded poorly to it, for no symptoms improved. Moreover, sensory ataxia worsened, suggesting the disease was progressing. Combined with the subacute onset, nearly two-month duration of the disease, and the positive anti-NF155 antibodies, the final diagnosis was anti-NF155 positive AN. He received a one-time dose of 500mg rituximab on H10, leading to a slight improvement in limb weakness. The muscle strength of the distal limbs of both upper limbs recovered to grade 4+, and the distal limbs of both lower limbs recovered to grade 5. Nevertheless, the mRS remained at 2. One month after discharge, we followed up with him by telephone and learned that walking instability still existed. He was admitted to another hospital and was treated with plasma exchange, after which the anti-NF155 titers of serum decreased from 1:320 to 1:10. But the symptoms did not relieve. The second telephone follow-up was made in the third month after his discharge from our hospital, and his symptoms as well as mRS score, remained stable.

## Discussion

The clinical features of patients with anti-NF155 antibodies AN differ from typical CIDP: younger at onset, a subacute or chronic disease course, distal weakness, sensory ataxia, tremor, and no or poor response to IVIg treatment (3, 8, 9). And the clinical feature of the case we report here is consistent with the prior studies but lacks tremor.

There have been few reports of AN with IgG4 anti-NF155 antibodies positive in CSF. One recent study on AN identified 3 patients with anti-NF155 antibodies positive in CSF. Subtype analysis and titration revealed that the antibodies were IgG4, and their titers were significantly lower than in serum (10). These findings are in agreement with the case we report. The presence of anti-NF155 antibodies in CSF of our case is confirmed by cell-based assay at titers much lower than the serum antibodies. No evidence of central demyelination was found, as his cerebral MRI and VEP were normal. Combined with the high protein content in CSF and hypertrophy of cervical and lumbar plexus, it is reasonable to assume that anti-NF155 antibodies appear in the CSF due to an inflammatory response leading to disruption of the blood-brain barrier and thus the entry of serum antibodies into the CNS. The possibility of intrathecal synthesis also cannot be excluded. But regrettably, the detection of oligoclonal bands was not performed. On the other hand, IgG anti-GD1b antibodies also seem to play a role in this patient’s clinical presentation, especially in ataxia, as it has been discovered that it may induce ataxia (11). The EBV antibodies may suggest a previous infection. However, this association with the current disease is unclear. Because we have no more evidence to confirm it and we are unable to follow up on the patient’s EBV antibodies.

A few points presented in our case may be instructive. First, the clinical features of this patient were mostly consistent with previous reports, except for the absence of tremors, which reflects the heterogeneity of this disease. The symptoms were partially relieved after treatment, implying that the utility of therapeutic strategies may require more extensive and dedicated studies. But also, it could be because of our not long enough follow-ups, as one study mentioned that the rituximab effect was not clearly seen until the third month of the disease (10). Second, the presentation of our case may suggest that anti-NF155 antibodies in CSF indicate more severe inflammation of the peripheral nerves than in an adverse patient, leading to more severe clinical features. Additional studies are needed to clarify whether antibodies in CSF are associated with a worse response to treatment. Third, given the pathogenicity of anti-GD1b antibodies (11), it seems reasonable to consider this as another cause of severe clinical features in this patient.

In conclusion, this patient presented a typical clinical feature of AN with serum and CSF anti-NF155 antibodies coexistent but poor response to IVIg, rituximab and plasma exchange. Prospective studies are necessary to demonstrate the potential role of anti-NF155 antibodies in CSF and help further understand this complex and heterogeneous disease.

## Data availability statement

The original contributions presented in the study are included in the article/supplementary material. Further inquiries can be directed to the corresponding author.

## Ethics statement

Written informed consent was obtained from the individual(s) for the publication of any potentially identifiable images or data included in this article.

## Author contributions

WW, QM contributed to conception and design of the study. WW organized the database and wrote the first draft of the manuscript. WW, LL, QM revised the manuscript. MZ, RY, DL, SY contributed the statistical analysis. All authors contributed to manuscript revision, read, and approved the submitted version.

## Funding

Yunnan Health Training Project of High-level Talents. Grant number: L-2017013.

## Conflict of interest

The authors declare that the research was conducted in the absence of any commercial or financial relationships that could be construed as a potential conflict of interest.

## Publisher’s note

All claims expressed in this article are solely those of the authors and do not necessarily represent those of their affiliated organizations, or those of the publisher, the editors and the reviewers. Any product that may be evaluated in this article, or claim that may be made by its manufacturer, is not guaranteed or endorsed by the publisher.
